# Neuronal life or death linked to depression treatment: the interplay between drugs and their stress-related outcomes relate to single or combined drug therapies

**DOI:** 10.1007/s10495-019-01557-5

**Published:** 2019-07-05

**Authors:** Przemyslaw Solek, Oliwia Koszla, Jennifer Mytych, Joanna Badura, Zaneta Chelminiak, Magdalena Cuprys, Joanna Fraczek, Anna Tabecka-Lonczynska, Marek Koziorowski

**Affiliations:** 1 Department of Animal Physiology and Reproduction, Faculty of Biotechnology, University of Rzeszow, Werynia 502, 36-100 Kolbuszowa, Poland; 2 Present Address: Department of Synthesis and Chemical Technology of Pharmaceutical Substances with Computer Modeling Lab, Faculty of Pharmacy with Division of Medical Analytics, Medical University of Lublin, Chodzki 4A, 20-093 Lublin, Poland

**Keywords:** Depression, Neurons, Antidepressants, Amitriptyline, Imipramine, Fluoxetine

## Abstract

Depression is a serious medical condition, typically treated by antidepressants. Conventional monotherapy can be effective only in 60–80% of patients, thus modern psychiatry deals with the challenge of new methods development. At the same moment, interactions between antidepressants and the occurrence of potential side effects raise serious concerns, which are even more exacerbated by the lack of relevant data on exact molecular mechanisms. Therefore, the aims of the study were to provide up-to-date information on the relative mechanisms of action of single antidepressants and their combinations. In this study, we evaluated the effect of single and combined antidepressants administration on mouse hippocampal neurons after 48 and 96 h in terms of cellular and biochemical features in vitro. We show for the first time that co-treatment with amitriptyline/imipramine + fluoxetine initiates in cells adaptation mechanisms which allow cells to adjust to stress and finally exerts less toxic events than in cells treated with single antidepressants. Antidepressants treatment induces in neuronal cells oxidative and nitrosative stress, which leads to micronuclei and double-strand DNA brakes formation. At this point, two different mechanistic events are initiated in cells treated with single and combined antidepressants. Single antidepressants (amitriptyline, imipramine or fluoxetine) activate cell cycle arrest resulting in proliferation inhibition. On the other hand, treatment with combined antidepressants (amitriptyline/imipramine + fluoxetine) initiates p16-dependent cell cycle arrest, overexpression of telomere maintenance proteins and finally restoration of proliferation. In conclusion, our findings may pave the way to better understanding of the stress-related effects on neurons associated with mono- and combined therapy with antidepressants.

## Introduction

According to the World Health Organization, depression is the most common mental disorder highly prevalent among millions of people across the world. It is also one of the main types of affective disorders and refers to a wide range of mental, cognitive, physical and behavioral health problems having a destructive impact on quality of everyday life [1–3].

Drugs available on the present pharmaceutical market are divided into groups according to their chemical structure and mechanism of action [4, 5]. Furthermore, antidepressants use is often associated with a long time administration, therefore their side effects and their wide range posses a significant risk [4, 6]. Also, their effectiveness is still questioned. It is now accepted that conventional monopharmacotherapy (with the use of single antidepressant) can be effective only in 60–80% of patients [7, 8]. For this reason, development of new and definitely better drugs is undoubtedly a huge challenge for modern psychiatry [9]. Research suggest that for many people with major depression, one drug is not enough to deal with a comprehensive range of symptoms experienced by patients. In case of treatment failure, the alternative therapies can be applied. These include among others combined administration of antidepressants with improved clinical efficacy [10, 11]. The use of combined therapy (the use of two or even more antidepressants with different mechanisms of action) has been already described in some papers [12–15]. Typically, during monopharmacotherapy patients are prescribed fluoxetine at a dosage 20–80 mg/day, which gives steady-state plasma levels of ~ 0.6–1 µM after 2–4 weeks of 60 mg/day [16]. Amitriptyline is usually prescribed 75–150 mg/day, which corresponds to 0.18–0.5 µM levels in plasma after 6 weeks of treatment [17]. In dual therapies, the doses can be lowered to 32.75 mg/day of both drugs with no changes in the effectiveness of the treatment [13]. Amitriptyline and fluoxetine are often considered as drugs of choice due to their neuroprotective and anti-inflammatory effects [18]. At the same moment, combining compounds with two different mechanisms of pharmacological action that complement each other in terms of the therapy efficacy raises concerns about the interactions between these drugs and the occurrence of potential adverse drug reaction [10].

For better understanding of antidepressants side effects, it is crucial to become acquainted with molecular mechanisms underlying their toxicity. Some reports indicate that antidepressants posses cyto- and genotoxic properties. Elmorsy et al. presented that fluoxetine, sertaline and clomipramine treatment reduces cellular oxygen consumption rates, activities of the mitochondrial complexes I and II and triggers an increase of lactate production. Moreover, higher concentrations of antidepressants were linked with upregulation of pro-apoptotic caspases-3, 8 and 9 in response to global reactive oxygen species-mediated DNA damage in rat primary blood barrier endothelial cells [19]. Further detailed studies revealed that structural chromosomal abnormalities [20], as well as telomeres shortening, may be involved in antidepressants-induced neurotoxicity [21]. As cited, the data on antidepressant-mediated toxicity is still fragmentary and mostly deals with the effect of monotherapy on stress-related effects.

Therefore, the specific aims of this study were to provide up-to-date comparative analysis on the relative neurotoxicity mechanisms of individual antidepressants (amitriptyline, imipramine, fluoxetine—‘single therapies’) and their combinations (amitriptyline/fluoxetine, imipramine/ fluoxetine—‘dual therapies’). Amitriptyline, imipramine and fluoxetine as considered to posses neuroprotective and anti-inflammatory characteristics were chosen for analyses. The study was designed to cover therapeutic, hyper-therapeutic and overdose concentrations and allowed us to determine the molecular mechanisms that drive drug-induced neurotoxicity within a 48 and 96 h time-frame.

## Materials and methods

### General

All reagents were purchased from Sigma-Aldrich (Poland), BioShop (Canada), Thermo Fisher Scientific (USA) or Chempur (Poland), had analytical grade purity and were used as obtained unless otherwise stated. All reactions, except cell culture, were carried out under normal atmospheric conditions at room temperature. All presented photos were not subjected to any image processing and represent raw data.

### Antibodies

Antibodies used were: anti-β-actin (#PA1-16889), anti-p16 (#PA5-16639), anti-p21 (#PA5-701151), anti-p27 (#PA5-13254), anti-p53 (#700439), anti-phospho-NF-κB (#PA5-37658), anti-NuMA (#PA132451), anti-calnexin (#MA3-027) (Thermo Fisher Scientific), anti-Bcl-2 (#sc-7382) (Santa Cruz), anti-active caspase 3 (#NB100-56113) (Novus Biologicals), anti-γH2AX (#CS208203) (Merck Millipore). Secondary HRP-conjugated: anti-mouse (#A9044) and anti-rabbit (#A0545) (Sigma).

### Cell culture conditions

The mouse hippocampal neuronal cells, HT-22, were purchased from Thermo Fisher Scientific, USA and routinely maintained in originally formulated Dulbecco's modified Eagle's medium (DMEM, Corning, USA) with high glucose (4.5 g/l) and sodium pyruvate (1 mM). The medium was supplemented with 10% heat-inactivated Fetal Bovine Serum (FBS, Biowest, France), 100 units/ml penicillin, 100 µg/ml streptomycin and 29.2 mg/ml l-glutamine. Neuronal cells were kept in a humidified CO_2_ incubator (New Brunswick Galaxy 170R, Thermo Fisher Scientific) at 37 °C with a mix gas containing 5% CO_2_ environment to maintain physiological pH. HT-22 were passaged every 3 days at 90% confluence by trypsinization with 0.25% trypsin/0.02% EDTA (Thermo Fisher Scientific, USA). For all procedures, cells were seeded in a constant density of 3.0 × 10^3^/cm^2^ 24 h before antidepressants treatment.

### Antidepressants treatment

The antidepressant drugs: (1) amitriptyline hydrochloride, (2) imipramine hydrochloride, (3) fluoxetine hydrochloride were purchased from Sigma-Aldrich and were dissolved in dimethyl sulfoxide (DMSO) to a 100 mM stock solution according to characteristic solubility. The drugs stock solutions were diluted in complete DMEM immediately before use and added to cells for 48 and 96 h.

### MTT assay

The MTT colorimetric method of cell proliferation and cytotoxicity determination was performed for the measurement of cell growth in response to antidepressants treatment. HT-22 cell cultures were prepared in 96-well format plates. The MTT stock solution was prepared in a physiologically balanced solution (1 × PBS) and added to each well at a final concentration of 5 mg/ml. After 4 h incubation at 37 °C in culture hood, the formazan crystals were dissolved with DMSO and the absorbance was measured at 590 nm (absorbance) and 620 nm (reference) wavelengths using PerkinElmer Victor X4 2030 microplate reader. The results are presented as %, while untreated CTRL cells were considered as 100%. IC_10_, IC_25_, IC_50_, IC_75_ and IC_90_ values were calculated by fitting linear regression equation, which was best suitable.

### Alterations in cellular morphology

We evaluated alterations in cell morphology in both, antidepressants-treated and control HT-22 cells after 48 and 96 h using an inverted light microscope Zeiss Axiovert 40 CFL equipped with a Zeiss AxioCam MRm3 camera (Carl Zeiss, Germany). Digital images were captured using phase contrast and AxioVs40 V 4.8.1.0 software.

### Determination of oxidative and nitrosative stress parameters

Measurement of intracellular superoxide radical anion was evaluated using fluorogenic DHE probe (dihydroethidium; at final concentration 5 µM, Thermo Fisher Scientific), nitric oxide (NO) production was measured using DAF-FM probe (4-amino-5 methyloamino-2’,7’-difluorofluorescein diacetate: at final concentration 5 µM, Cayman Chemical) while reduced glutathione levels were estimated using robust intracellular thiol probe for GSH (Thiol tracker violet: at final concentration 5 µM, Thermo Fisher Scientific). Digital images and quantifications were performed using InCell Analyzer 2000 and presented as relative fluorescence units (RFU). A minimum of 1000 cells were counted in each sample.

### ATP-luminescence measurements

Total ATP cellular levels were detected using ATP-luminescence system (PerkinElmer, USA) for the quantitative evaluation of proliferation and antidepressants-mediated cytotoxicity of cultured HT-22 cells, according to the manufacturer’s instructions. In brief, the lyophilized substrate solution vial was resolved in adequate volume of substrate buffer solution. Then, mammalian cell lysis solution was added and cells were incubated for 5 min in an orbital shaker. After incubation, the substrate solution was added to each well and mixed again in an orbital shaker. The luminescence was measured at dark-adapted plate using PerkinElmer Victor X4 2030 microplate reader. The values were given as relative values to control cells.

### Analysis of cell cycle profile and micronuclei formation

The cell cycle profile analysis by DNA content measurement was performed using two fluorescent dyes (nuclei: DAPI at final concentration 1 µg/ml; cell cytoplasm: Calcein-AM at final concentration 0.3 µg/ml). Images were captured using InCell Analyzer 2000 and the output was processed by the ImageJ software with the DNA cell cycle plug-in. Cell cycle phases in a given cell population were presented as % of cells in G0/G1, S and G2/M phases (cell cycle distribution) or as % of micronuclei positive cells (micronuclei formation). A minimum of 1000 cells were counted in each sample.

### Immunofluorescence staining of γH2AX

The phosphorylation status of γH2AX as a biomarker of DNA damage was detected using immunostaining standard protocol. In short, cells were fixed in 4% paraformaldehyde, permeabilized in PBS containing 0.25% Triton X-100, blocked with 1% BSA and then incubated with the primary antibody prepared in 1% BSA in PBST overnight at 4 °C. Next, the cells were washed three times in PBS (5 min each wash) and nuclei were visualized with Hoechst 33258. Fluorescent images were captured using InCell Analyzer 2000 and ImageJ software was applied for γH2AX quantification.

### Western blot

Total protein extracts were prepared and Western Blot method was carried out as previously described in Solek et al with minor modifications [22]. Briefly, 30 µg of whole cell protein lysates were separated by size using % SDS-PAGE electrophoresis. After electrophoresis, the separated proteins were electroblotted onto a methanol-activated polyvinylidene difluoride membranes (PVDF, Thermo Fisher Scientific) and blocked in 1% BSA for 1 h to prevent nonspecific binding of the antibody probes. The proteins were then complexed with specific primary and secondary HRP-conjugated antibodies. Detection and localization of target proteins were performed using ECL western blotting substrate (BioRad, USA) and Fusion Fx7 system (Viber Lourant) according to the provided instructions. The relative protein expression levels were normalized to β-actin (GelQuantNET software).

### Presentation of results and statistical analysis

Adobe Photoshop CC software was used to process images and to design all figures. Data shown represent the means ± standard deviation. The experiments were repeated three times with at least n = 3 per treatment condition. Statistical multiple comparisons were performed using GraphPad Prism ver. 6.0 and the data were assessed with one-way ANOVA followed by Dunnett post hoc test. A p-value of < 0.05 was considered statistically significant between groups and are displayed as: *^/^^p < 0.05; **^/^^^p < 0.01; ***^/^^^^p < 0.001. Asterisks (*) indicate the comparison between CTRL (non-treated) and antidepressants-treated cells while carets (^^^) indicate the comparison between the same drugs in different periods (48 h, 96 h).

## Results

### Antidepressants regulate the neuronal mitochondrial activity of mouse hippocampal cells

**Fig. 1:**
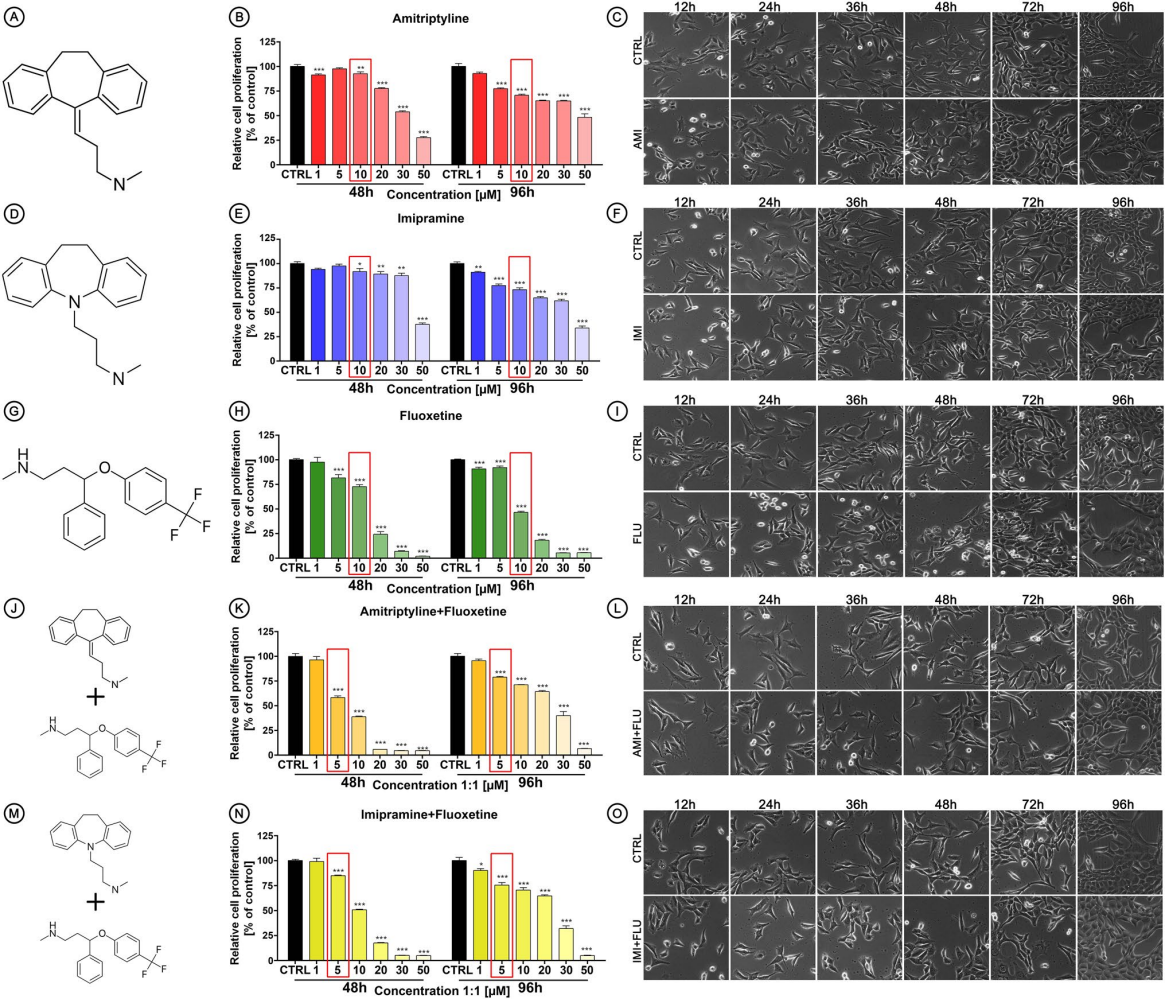
Antidepressants-mediated effects in mouse hippocampal neurons (HT-22 cells). Chemical structure of amitriptyline (**a**), imipramine (**d**), fluoxetine (**g**), amitriptyline + fluoxetine (**j**), imipramine + fluoxetine (**m**). Cells were treated for 48 and 96 h with wide range of antidepressants concentrations and MTT assay was performed (**b**, **e**, **h**, **k**, **n**) to chose one concentration for further studies and then the effects of single 10 µM and combined 5 + 5 µM antidepressants on morphological characteristics (**c**, **f**, **i**, **l**, **o**) were evaluated. Magnification of the objective lens × 10. Bars indicate SD, n = 3, ***^/^^^^p < 0.001, **^/^^^p < 0.01, *^/^^p < 0.05, no indication—no statistical significance (one-way ANOVA and Dunnett’s a posteriori test)

At the beginning, the HT-22 cells were treated with amitriptyline (AMI) (Fig. 1a), imipramine (IMI) (Fig. 1d) and fluoxetine (FLU) (Fig. 1g) or co-treated with AMI + FLU (Fig. 1j) and IMI + FLU (Fig. 1m) with a wide concentrations range (1–50 µM). Then, the in vitro cytotoxicity after 48 and 96 h based on MTT assay was evaluated. We found that relative cell proliferation was reduced after antidepressants treatment in a concentration-dependent manner. The differences were less pronounced after 96 h than after 48 h in any experimental set, except FLU (Fig. 1b, e, h, k, n). ICs values for the observed effects generated via regression analyses are presented in Table 1.

**Table 1:** ICs (µM) of antidepressants (ADs) for HT-22 cells

ADs	Time of exposure (h)	IC_90_	IC_75_	IC_50_	IC_25_	IC_10_
AMI	48	63.33 ± 5.34	52.93 ± 4.64	35.59 ± 3.48	18.25 ± 2.34	7.85 ± 1.69
	96	94.20 ± 21.89	76.35 ± 18.50	46.59 ± 13.01	16.82 ± 8.05	1.03 ± 5.94
IMI	48	88.83 ± 9.47	74.49 ± 8.18	50.59 ± 6.14	26.68 ± 4.52	12.34 ± 3.95
	96	72.02 ± 9.59	58.62 ± 8.17	36.28 ± 5.93	13.94 ± 4.08	1.03 ± 3.43
FLU	48	37.26 ± 1.93	30.33 ± 3.08	18.80 ± 2.44	7.27 ± 2.86	0.34 ± 3.19
	96	36.57 ± 1.25	29.28 ± 1.25	17.14 ± 1.30	4.99 ± 1.42	2.29 ± 1.51
AMI + FLU [1:1]	48	34.29 ± 3.64	26.49 ± 3.26	13.50 ± 2.69	0.51 ± 2.26	0.16 ± 2.09
	96	47.96 ± 5.70	39.47 ± 5.28	25.32 ± 4.59	11.16 ± 3.93	1.03 ± 3.56
IMI + FLU [1:1]	48	34.47 ± 1.64	29.33 ± 1.39	17.45 ± 0.98	5.56 ± 0.67	1.57 ± 0.58
	96	47.62 ± 6.48	39.20 ± 6.14	25.17 ± 5.64	11.14 ± 5.20	1.03 ± 4.98

Based on cytotoxicity results, we chose one concentration of antidepressants for further research (marked in the red frames), due to the fact that high, toxic concentrations are not clinically applicable.

### Effects of antidepressant treatment on cell morphology

To investigate the effects of selected antidepressants on cell morphology, we treated mouse hippocampal cells for a period of 96 h. Single (10 µM concentration) or combined (5 µM + 5 µM concentration) treatment did not significantly alter the cell morphology. Cells were characterized by a typical neuron-like morphology, elongated shape, with a single nucleus and equally distributed and granular cytoplasm (Fig. 1c, f, l, o). Although no consistent morphologic abnormalities in almost all treated sets were observed, FLU-treated cells were altered in their growth characteristics. We observed round and shrunk cells with smooth plasma membrane as well as vacuolization and partial detachment from the substrate (Fig. 1i).

### Oxidative and nitrosative stress versus antioxidant defense

**Fig. 2:**
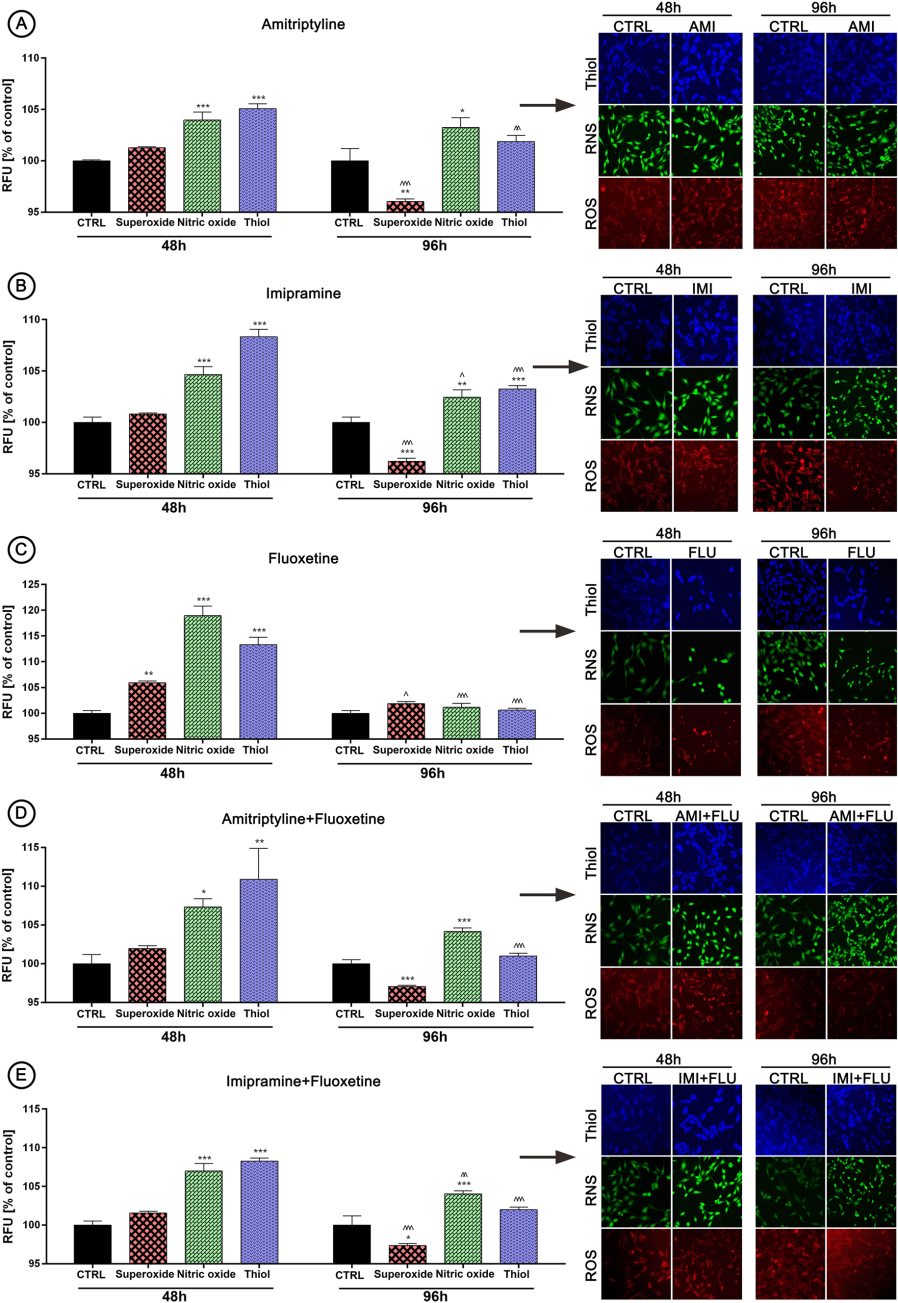
Antidepressants-mediated effects on oxidative/nitrosative stress and antioxidant defense. HT-22 cells were treated for 48 and 96 h and the effects of antidepressants on superoxide, nitric oxide and thiol production (**a**–**e**) were evaluated. Red fluorescence—dihydroethidium (ROS), green—4-amino-5-methylamino-2′,7′-difluoro-fluorescein diacetate (NO), blue fluorescence—Thiol Tracker (Thiol). Magnification of the objective lens × 10. Bars indicate SD, n = 3, ***^/^^^^p < 0.001, **^/^^^p < 0.01, *^/^^p < 0.05, no indication—no statistical significance (one-way ANOVA and Dunnett’s a posteriori test) (Color figure online)

Next, we decided to evaluate what is the cause of cell proliferation reduction, therefore the parameters of oxidative and nitrosative stress were controlled. Indeed, drugs caused an increase in the production of nitric oxide in each experimental set analyzed. Furthermore, we find out that antidepressants activated defense mechanisms against free radicals by thiol overproduction after 48 h, but not after 96 h. Interestingly, the superoxide level remained at the control level after 48 h, but we observed an unambiguous decline in the superoxide production after 96 h (again as before—except FLU) (Fig. 2a–e).

### Mitochondrial response to nitrosative and oxidative imbalance

**Fig. 3:**
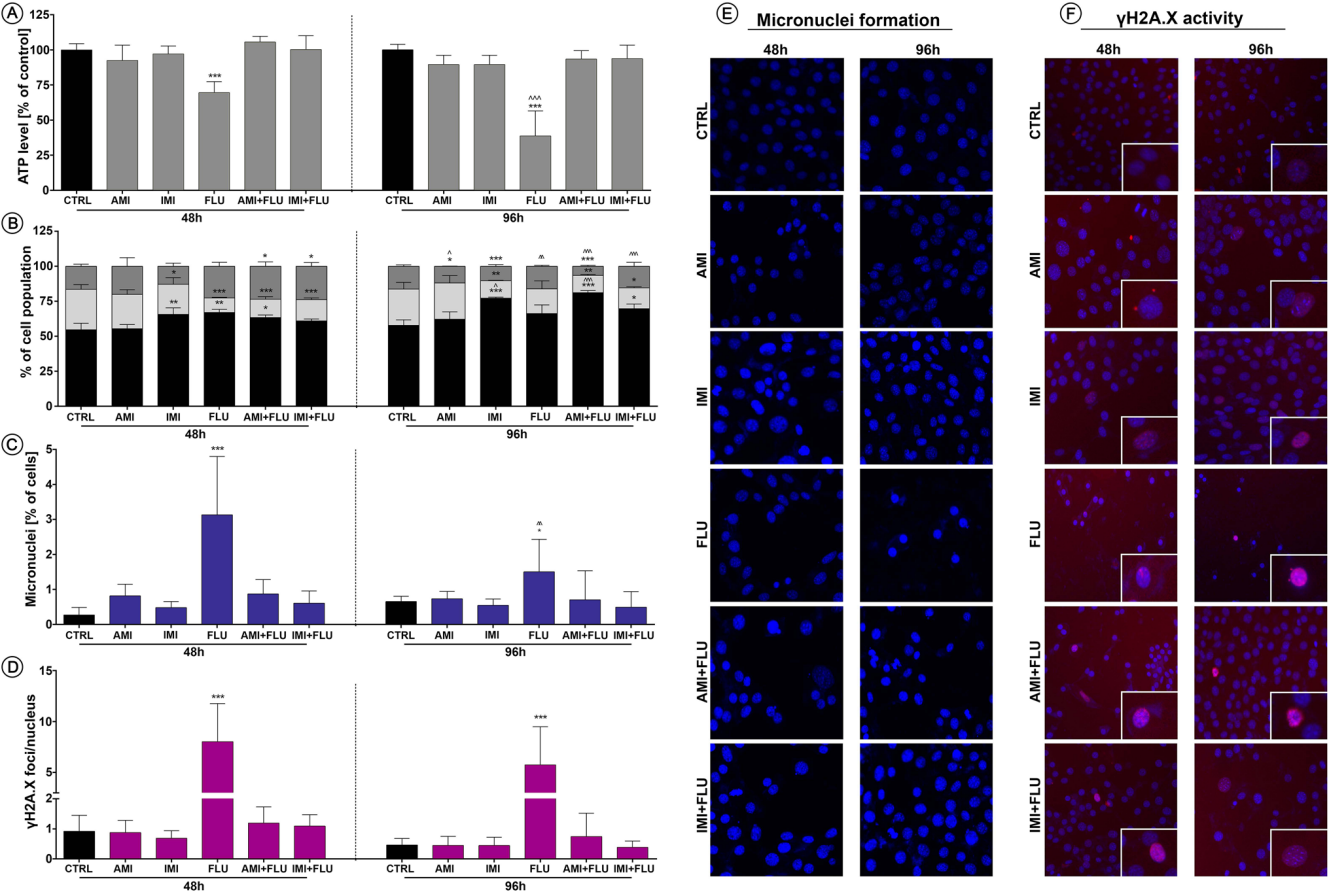
Stress-related effects of antidepressants on cellular and biochemical features. Mouse hippocampal neurons were treated for 48 and 96 h and then the effects of antidepressants on ATP generation (**a**), cell cycle progression (**b**), micronuclei generation (**c**), γH2AX formation (**d**) were evaluated. Representative images of micronuclei formation (**e**) and γH2AX activity (**f**) are shown. Blue fluorescence—Hoechst 33342, red—Texas Red. Magnification of the objective lens × 10. Bars indicate SD, n = 3, ***^/^^^^p < 0.001, **^/^^^p < 0.01, *^/^^p < 0.05, no indication—no statistical significance (one-way ANOVA and Dunnett’s a posteriori test) (Color figure online)

In the view of results above, we investigated the relationship between alterations in the oxidative and nitrosative imbalance and mitochondrial function. We noted moderately reduced intracellular ATP levels promoted by mitochondria damage (results not statistically significant). The effect was even more pronounced after 96 h as compared with control. In addition, the most significant declines were observed in the case of cells treated with FLU for both, 48 and 96 h (Fig. 3a).

### Interaction between DNA and antidepressants

Further, in order to explain these toxicities mechanisms, we studied the relationship between selected concentrations of the antidepressants and the cell cycle progression, micronuclei formation and DNA damage. We observed that antidepressant treatment induced statistically significant reduction in the G2/M or S cell cycle phase cell populations in all experimental sets after 48 h and the effect remained stable even after 96 h. Moreover, four antidepressants sets (except AMI) displayed similar increases in G0/G1 phase cell number (Fig. 3b). In turn, antidepressants promoted DNA damage response in all cell lines examined by an increase in the number of cells with micronucleus frequency correlated with DNA double-strand breaks (DSBs). Again, the differences were less pronounced after 96 hours when compared to 48 h (Fig. 3c–f).

### Cell-signaling pathways activation and biochemical response to antidepressants

**Fig. 4:**
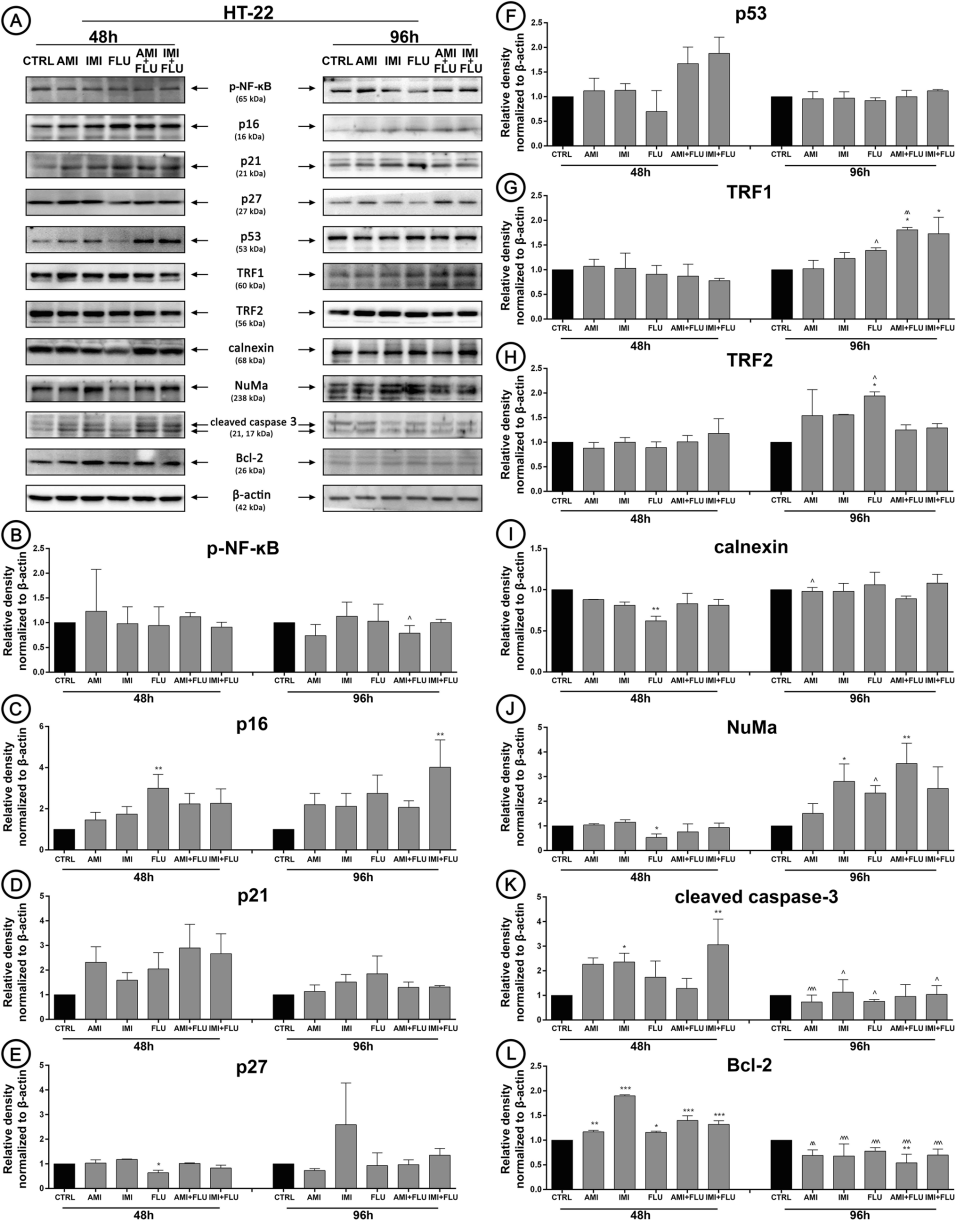
Antidepressants-mediated effect on cellular protein content. HT-22 cells were treated with antidepressants for 48 and 96 h and densitometry analysis of NF-κB (**b**), p16 (**c**), p21 (**d**), p27 (**e**), p53 (**f**), TRF1 (**g**), TRF2 (**h**), calnexin (**i**), NuMa (**j**), cleaved caspase 3 (**k**), Bcl-2 (**l**) was evaluated. Representative Western Blots are presented (**a**). Bars indicate SD, n = 3, ***^/^^^^p < 0.001, **^/^^^p < 0.01, *^/^^p < 0.05, no indication—no statistical significance (one-way ANOVA and Dunnett’s a posteriori test)

Finally, we wanted to elucidate the molecular mechanisms underlying the toxic effects of antidepressants (Fig. 4a). Generally, antidepressants treatment did not activate redox-sensitive transcription factor (as assessed by the evaluation of its phosphorylation status) NF-κB (Fig. 4b). In turn, the expression pattern of cell cycle regulatory proteins was affected. We noted up-regulated expression of DNA damage-associated protein p16 and p27 after FLU treatment (Fig. 4c, e) concomitant with the constant level of p21 and p53 (independent signaling pathways) (Fig. 4d, f). Based on these findings, we also focused our attention on proteins implicated in telomere length protection. Similarly, we did not detect significant changes in TRF1 and TRF2 after 48 h treatment, but interestingly, only FLU treatment resulted in upregulation of both proteins synthesis after 96 h (Fig. 4g, h). Continuing, in the case of FLU (48 h treatment), we observed downregulation of protein required for efficient folding of newly glycoproteins (calnexin) and protein essential for the formation of the mitotic spindle (NuMa) (Fig. 4i, j). Moreover, we confirmed activation of apoptotic cell death pathway in response to unrepairable DNA damage. We certainly confirmed that antidepressants treatment resulted in simultaneous upregulation of active caspase 3 and Bcl-2 after 48 h, while 96 h incubation caused active caspase 3-dependent Bcl-2 decrease (Fig. 4k, l).

## Discussion

Our study was designed to compare the possible toxic effects of single and dual antidepressants used in therapeutic, hyper-therapeutic and overdose concentrations after 48–96 h exposures.

In general, firstly we demonstrated a clearly concentration-dependent relationship between the concentration of antidepressants and decrease of cell metabolic activity what is in agreement with previous reports [23–26]. Here, we additionally provide evidence that the decrease in the relative cell proliferation in cells treated for 96 h is less pronounced than in cells treated for 48 h. Also, cells treated with combination of antidepressants better activate adaptation and repair mechanisms, what results in a significant acceleration of their growth rate when compared to cells treated with single antidepressants. The other authors suggest that a significant number of cells change characteristics in morphological shape and detach from the bottom of the culture flask at high drug concentrations which may indicate apoptosis [27, 28]. Interestingly in our research, only FLU exposure led to progressive morphological changes. However, we believe that morphological evaluation is questionable to draw far-reaching conclusions about the apoptosis, which is why we decided to carry out further studies to clarify the precise mechanisms of this process. Thus, in the next step, we were wondering what observed changes in metabolic activity could actually result from. This could be to due to initiation of oxidative and nitrosative stress, which in turn can directly or indirectly cause lipids, proteins and DNA damage [29]. As excepted, we observed a slight increase in the total ROS pool after 48 h, while longer incubation resulted in a significant decrease, probably due to the activation of enzymatic mechanisms of cell protection. Our results are consistent with other authors [30–32]. Further, we observed a significant increase in NO after 48 and 96 h incubation, similarly to others [33–37]. Taken together these data, we suppose that high concentration of RNS is perhaps responsible for further cellular and molecular events that we observed later. Interesting is fact, that observed effect at this point was comparable between cells treated with single and combined antidepressants.

Consequently, exposure to oxidative and nitrosative stress may be sufficient to impair ATP production, increase mitochondrial membrane permeability and further alter functions of mitochondria. Indeed, in this study, we noted a decrease in ATP after 48 h which was even more pronounced after 96 h in cells treated with single antidepressant, fluoxetine. At the same time, the combination of fluoxetine with amitriptyline or imipramine did not cause similar effects. Others also reported that antidepressants lower ATP production and inhibit mitochondrial complex activity which is essential for reuptake of Ca^2+^ into the endoplasmic reticulum [38, 39]. ATP reduction was accompanied by decrease in G2/M or S cell cycle phase cell populations with simultaneous increase in G0/G1 phase cell number. At the same time, the pool of cells in G0/G1 phase in cells treated with combination of antidepressants for 96 h increased when compared to 48 h. The same phenomenon was not observed in cells treated with single antidepressants. This strongly suggests that cells treated with dual antidepressants activated mechanisms crucial for cellular repair and adaptation. The fact is that the cell cycle arrest at any of the checkpoint is thought to provide the cells time to repair the critical damage before DNA replication [40]. We observed an increase in micronuclei frequency correlated with DNA double-strand breaks after ADs treatment. Interesting fact is that these incidents were observed in cells treated with fluoxetine but not in cells treated with fluoxetine with amitriptyline or imipramine. Also, due to the longer incubation the level of observed DNA damage significantly has lowered. Other studies confirm that fluoxetine blocks the cell cycle in the G0/G1 phase due to a disturbance of the skp2-CKS1 complex, which is required for the proper course of the cell cycle [41]. The arrest can also be correlated with a reduction of cyclin A protein expression, responsible for regulating cell division [42, 43].

Once we had identified that ADs possessed activity to disturb the balance in the antioxidant systems, impair ATP production, cell cycle deregulation as well as H2AX phosphorylation and MN formation, we turned our attention to understanding their mechanism of action. We noted a slight but not significant decrease in phospho-NF-κB level in HT-22 cells. Other studies confirm that drugs cause a drop in NF-κB protein activity, which is closely correlated with a relatively low level of free oxygen radicals [44, 45]. The first mechanistic explanation of observed events is the observed up-regulation in p16 and p27 protein pools. p27 protein regulates cell cycle arrest in G1/G0 phase and is responsible for induction of programmed cell death, therefore, we assume that fluoxetine, amitriptyline and imipramine alone in the studied concentration lead to increased apoptosis. At the same time, there was no activation of p21 and p53 dependent pathways. To be precise, the p53 protein may be responsible for the prolongation of the G1 phase, which allows the repair of DNA damage [46]. On the other hand, the p16 protein also controls the normal progression of the cell cycle. Moreover, p16 over-expression occurs when numerous DNA damage and replication errors appear, which results in the cell cycle arrest. Here, we observed increased expression of p16 protein after combined antidepressants treatment, which confirms the prolonged inhibition of the cell cycle in the G0/G1 phase to allow cells to repair the damage. Furthermore, a high expression of p16 can also suggest damage in telomere sections. In fact, we noted up-regulation of both telomere maintenance proteins, TRF1 and TRF2 after 96 h treatment (AMI/IMI + FLU co-treatment). Further, it was hypothesized that ADs treatment increases apoptotic cell death via caspase-3 pathways activation in a concentration-dependent manner [5, 47, 48]. Noteworthy, in the present study, a slight increase in the caspase-3 expression level due to the ADs treatment was observed only after 48 h in cells treated with single antidepressants. This effect was not reported in cells treated with combination of antidepressants. This demonstrates that apoptosis is activated in the most fragile cells and is associated with dysfunction of adaptive mechanisms. In the regulation of the caspase function proapoptotic and antiapoptotic proteins from the Bcl-2 family are also involved. What's more, the neuroprotective properties of Bcl-2 protein are known [49, 50] but the evidence suggest that drugs in a concentration-related manner may be neuroprotective to hippocampal neurons [51]. Our data indicated an upregulation of Bcl-2 expression after 48 h. It seems that the use of antidepressants changes the level of Bcl-2 probably associated with the survival of neurons. Thus, in this study the reduction of cell number observed in cells treated with single antidepressants results rather from the proliferation inhibition rather than apoptosis initiation. On the other hand, acceleration of proliferation observed in cells co-treated with two antidepressants results from adaptation of effective adaptation and repair mechanisms.

**Fig. 5:**
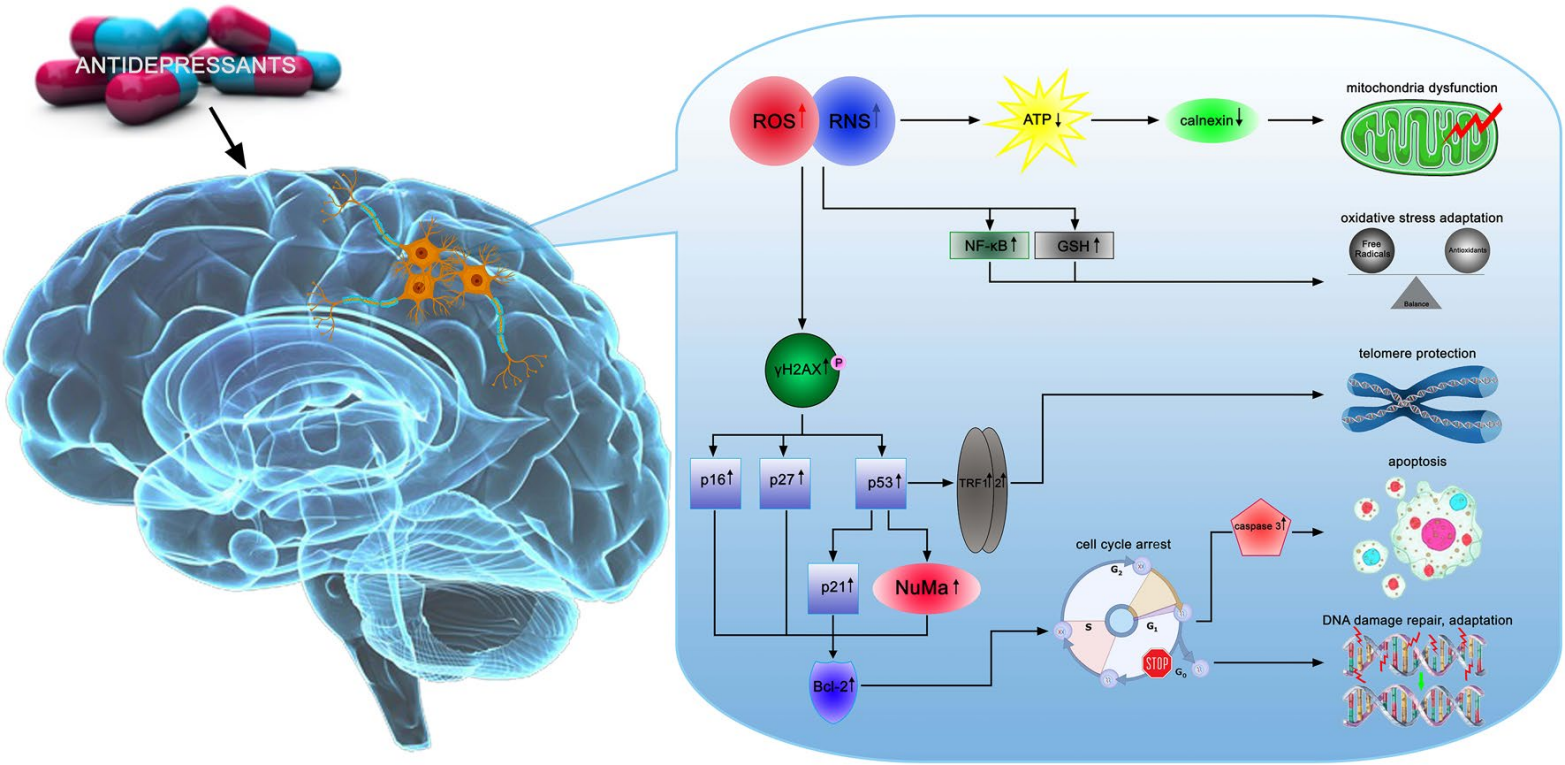
Proposed molecular mechanisms underlying the complex and multi-stage processes of hippocampal cell response to antidepressants

Mitochondrial ROS production is often found to increase with ER stress [52]. Based on these findings it remains to be determined how the antidepressants may affect calnexin, protein involved in apoptosis triggered by endoplasmic reticulum stress [53] and NuMa involved in major cellular events such as DNA damage response, apoptosis and p53-mediated growth-arrest [54]. Indeed, we observed up-regulated synthesis of NuMa protein only after 96 h exposure in cells treated with combination of antidepressants. We suppose that this protein is involved in long-term adaptation and p16-dependent cell cycle arrest but at the same time independent of p53. In turn, we noted down-regulation of calnexin which once again proves the positive action of the repair mechanisms activation (Fig. 5).

In conclusion, we provide for the first time evidence that co-treatment with amitriptyline/imipramine + fluoxetine initiates in cells adaptation mechanisms associated with TRF1/TRF2 proteins which adjust cells to stress and finally exerts less genotoxic events than in cells treated with single antidepressants.
